# Surfaceome Profiling of Rhabdomyosarcoma Reveals B7-H3 as a Mediator of Immune Evasion

**DOI:** 10.3390/cancers13184528

**Published:** 2021-09-09

**Authors:** Roxane R. Lavoie, Patricio C. Gargollo, Mohamed E. Ahmed, Yohan Kim, Emily Baer, Doris A. Phelps, Cristine M. Charlesworth, Benjamin J. Madden, Liguo Wang, Peter J. Houghton, John Cheville, Haidong Dong, Candace F. Granberg, Fabrice Lucien

**Affiliations:** 1 Department of Urology, Mayo Clinic, Rochester, MN 55902, USA; r.lavoie.roxane@mayo.edu (R.R.L.); gargollo.patricio@mayo.edu (P.C.G.); mohamed.ahmed@mayo.edu (M.E.A.); kim.yohan@mayo.edu (Y.K.); Emily.E.Baer@dmu.edu (E.B.); dong.haidong@mayo.edu (H.D.); granberg.candace@mayo.edu (C.F.G.); 2 Greehey Children’s Cancer Research Institute, San Antonio, TX 78229, USA; PhelpsD@uthscsa.edu (D.A.P.); houghtonp@uthscsa.edu (P.J.H.); 3 Proteomic Core, Mayo Clinic, Rochester, MN 55902, USA; charlesworth.cristine@mayo.edu (C.M.C.); madden.benjamin@mayo.edu (B.J.M.); 4 Division of Computational Biology, Mayo Clinic, Rochester, MN 55902, USA; Wang.Liguo@mayo.edu; 5 Department of Anatomic Pathology, Mayo Clinic, Rochester, MN 55902, USA; Cheville.John@mayo.edu; 6 Department of Immunology, Mayo Clinic, Rochester, MN 55902, USA

**Keywords:** rhabdomyosarcoma, targeted therapies, cell surface proteomics, B7-H3

## Abstract

**Simple Summary:**

Rhabdomyosarcoma (RMS) is the most common soft-tissue sarcoma in children, and there is a critical need to develop efficacious and tolerable anticancer therapies against this aggressive disease. To uncover druggable RMS-associated tumor antigens, we analyzed the cell surface protein repertoire in RMS tumor cells and normal tissue. We identified several surface proteins highly enriched in RMS, including the immune checkpoint molecule B7-H3. A further analysis using patient specimens showed that B7-H3 is overexpressed in most of RMS tumors and weakly or not detected in normal organs. Interestingly, we found that B7-H3 depletion was associated with higher immune cell killing activity against tumor cells. In line with this, high B7-H3 tumor expression was associated with lower CD8 T-cell density. Our study reveals novel RMS-associated proteins for the development of targeted therapies. In addition, we demonstrate that targeting B7-H3 function can pave the way for the design of new immunotherapies in the treatment of RMS.

**Abstract:**

Novel therapeutic strategies are needed for the treatment of rhabdomyosarcoma (RMS), the most common soft-tissue sarcoma in children. By using a combination of cell surface proteomics and transcriptomic profiling of RMS and normal muscle, we generated a catalog of targetable cell surface proteins enriched in RMS tumors. Among the top candidates, we identified B7-H3 as the major immunoregulatory molecule expressed by RMS tumors. By using a large cohort of tissue specimens, we demonstrated that B7-H3 is expressed in a majority of RMS tumors while not detected in normal human tissues. Through a deconvolution analysis of the RMS tumor RNA-seq data, we showed that B7-H3-rich tumors are enriched in macrophages M1, NK cells, and depleted in CD8^+^-T cells. Furthermore, in vitro functional assays showed that B7-H3 knockout in RMS tumor cells increases T-cell mediated cytotoxicity. Altogether, our study uncovers new potential targets for the treatment of RMS and provides the first biological insights into the role of B7-H3 in RMS biology, paving the way for the development of next-generation immunotherapies.

## 1. Introduction

Rhabdomyosarcoma (RMS) is the most common childhood soft tissue sarcoma, with nearly 20% of patients presenting with locally aggressive and/or metastatic disease [1]. RMS is classified into two molecular subtypes based on the expression of the fusion gene *PAX3/7 FOXO1* [1,2]. Patients with fusion-positive RMS usually have a poorer outcome compared to fusion-negative RMS [3]. The *PAX3/7-FOXO1* fusion protein drives an oncogenic transcriptional program, including the upregulation of genes involved in invasion, proliferation, and survival [4]. In contrast, fusion-negative RMS does not harbor *PAX3/7-FOXO1* fusion but a variety of mutations on oncogenes, including *RAS*, *PIK3CA*, *FGFR4*, and *TP53* [5]. Multimodal therapies, including surgery, radiotherapy, and chemotherapy, have been the standard care for the treatment of RMS. The current chemotherapy regimens consist of combinations of Vincristine, Dactinomycin, and Cyclophosphamide and have shown to improve the survival rates by 60–90% in patients with localized disease [6,7,8]. In contrast, 90% of RMS-related deaths occur within two years following diagnosis and are mostly related to disease recurrence. Importantly, long-term effects and life-threatening complications often occur in the lifetime of RMS survivors [9,10]. This underscores the need for effective and more tolerable therapies for the treatment of RMS.

Antibody-based therapies, including immune checkpoint inhibitors, bispecific antibodies, antibody–drug conjugates, and CAR-T therapy, have transformed the therapeutic landscape of adult cancers [11]. Antibody-based therapies have a common denominator with the specific binding of a protein expressed on the surface of tumor cells. Cell surface proteins are ideal targets for anticancer therapies as their accessible extracellular domain makes pharmacological interventions more effective. To date, cell surface proteins account for ~60% of all FDA-approved drugs [11]. Furthermore, it provides an alternative strategy to treat tumors with undruggable intracellular oncogenic alterations that drive transcriptional and translational programs, resulting in the aberrant expression of druggable cell surface proteins [12,13,14]. The surface proteome is composed of cell adhesion molecules; nutrients; metabolite transporters; immunoregulatory molecules; and growth factor receptors that control cancer cell behaviors, aggressiveness, and their response to therapy. The identification of tumor-associated surface proteins may provide important insights into tumor biology and be further exploited as therapeutic vulnerabilities.

In this study, we employed cell surface proteomics (surfaceomics) to establish a comprehensive map of cell surface proteins expressed in RMS. By integrating the transcriptomic data and proteomic data of RMS tumors and normal tissue, we revealed an RMS-specific cell surface protein signature. Among the top upregulated proteins, we identified B7-H3 as the major immunoregulatory molecule expressed in RMS. We found that B7-H3 mediates tumor immune evasion through functional assays and transcriptomic characterization of the tumor immune landscape of RMS tumors. Our findings support B7-H3 as a therapeutic target for antibody-based therapies and provide new biological insights on its immunomodulatory role in RMS. Finally, this study demonstrates the potential of surfaceomics for cancer research and drug development.

## 2. Materials and Methods

### 2.1. Cells and Reagents

Human pediatric rhabdomyosarcoma cell lines SJCRH30 (RH30) (alveolar rhabdomyosarcoma (aRMS) fusion-positive, cat# CRL-2061) and RD (embryonal RMS (eRMS), fusion-negative, cat# CCL-136) were purchased from ATCC (Manassas, Virginia, USA). Two normal human skeletal muscle cell lines were purchased from Lonza (Basel Switzerland) (cat#PCS-950-010) and ATCC (cat#CC-2561). The RMS cell lines RH36 (fusion-negative) and RH18 (fusion-positive) were obtained from Dr. Peter Houghton. The RH30 cell line was grown in RPMI-1640 with 10% FBS (Gibco, Waltham, MA, USA) and 1-mg/mL Pen–Strep (Gibco), the RD cell line was grown in DMEM with 10% FBS (Gibco) and 1-mg/mL Pen–Strep (Gibco), and the normal muscle cell lines were cultured in HSkMC Growth Medium (Cell Applications, cat#151-500, San Diego, CA, USA). The RMS-MC02 primary cells were isolated from a resected embryonal (fusion-negative) RMS tumor (IRB #16-006956) and cultured in DMEM-F12 (Gibco) with 10% FBS (Gibco) and 1-mg/mL Pen–Strep (Gibco). The B7-H3 knockout RH30 cell line was generated using CRISPR/Cas-9 (Santa Cruz Biotechnology, sc-402032, Dallas, TX, USA), and negative selection was performed by flow cytometry using anti-B7H3 PE (DCN.70 clone, BioLegend, cat#331606, San Diego, CA, USA). All cell lines were maintained in an incubator with a humidified atmosphere and 5% CO_2_ at 37 °C.

### 2.2. Cell surface Biotinylation and Mass Spectrometry

A total of 5 × 10^7^ cells from each cell line were subjected to cell surface biotinylation. EZ-Link-Sulfo-NHS-SS-biotin (Thermo Scientific, cat#21331, San Diego, CA, USA) was added to cultured cells for 30 min at 4 °C. The cells were washed with 50 mM of glycine to quench the unbound biotin. The cells were lysed in NP-40 lysis buffer, and the biotinylated cell surface proteins were affinity-purified on streptavidin magnetic beads (Thermo Scientific, cat#88816). After stripping off the nonspecifically bound proteins by several rounds of washing with the lysis buffer, the labeled proteins were reduced with 10-mM TCEP (Thermo Scientific, cat#77720) for 30 min at 50 °C and followed by alkylation with iodoacetamide (Thermo Scientific, 90034) for 30 min in the dark at room temperature. The proteins were run in a SDS-PAGE electrophoresis gel and submitted for mass spectrometry (additional information in the Supplementary Materials) [15,16,17].

### 2.3. Bioinformatic Annotation of Cell Surface Proteins

Genes encoding for the cell surface proteins were assembled from Gene Ontology GO:0005886 (plasma membrane) and UniProt using the keywords “homo sapiens” and “single-pass transmembrane domain” and “multi-pass transmembrane domain”.

### 2.4. Expression Analysis of RMS-Enriched Cell Surface Proteins in RMS Tumors and Normal Tissue

The RNA sequencing data was processed and analyzed as previously reported [5] (GEO: GSE108022). The gene-level raw read counts matrix file was downloaded from the GEO database, and differential expression analyses were performed using DESeq2 [18]. Specifically, the DESeq2 default parameters/methods were used to estimate the size factors, estimate the dispersion, and perform the statistical tests. The cell surface proteins differently expressed between RMS (*n* = 101) and normal muscles (*n* = 5), fusion-negative (*n* = 66) and normal muscles, and fusion-positive (*n* = 35) and normal muscles with a fold change ≥ two and adjusted *p*-value < 0.05 were undertaken for further analysis. To analyze the normal tissue expression of the RMS-enriched cell surface proteins, tissue-specific transcriptomes and proteomes were obtained from Jiang, L et al. that consisted of transcriptomic and proteomic analyses of 201 samples from 32 tissue types of 14 normal individuals [19]. The median relative protein and RNA abundances for each tissue type were transformed into absolute values (reversed log2) for further analysis. To identify the best therapeutic candidates, a composite ranking of the cell surface proteins was generated, where the cell surface proteins were ranked with equal weights based on their RNA and protein expression in the RMS tumors and normal tissue. For tumor expression, the genes and proteins were ranked from the most expressed to the least expressed. For normal expression, the genes and proteins were ranked from the least expressed to the most expressed.

### 2.5. Deconvolution Analysis of Bulk RNA-seq

From the GSE108022 RNA sequencing data, the genes with an average RPKM value across all samples less than 0.5 were removed, and then, the fraction of cell types (B cells, M1 macrophages, M2 macrophages, monocytes neutrophils, NK cells, CD4 T cells, CD8 T cells, Tregs, and dendritic cells) were identified and estimated using the QuanTIseq pipeline [20]. The RMS tumors were stratified by B7-H3 expression (25% highest and 25% lowest), and the immune cell abundance was compared between both groups.

### 2.6. T-Cell Cytotoxicity Assay

Adherent RH30 wild-type and B7-H3KO cells were plated in a 96-well plate and incubated 30 min at 37 °C with calcein-AM (Biolegend, 425201). The cells were then washed twice with Live Cell Imaging Solution (Gibco, A14291DJ) and kept in RPMI-1640 (no phenol red) supplemented with 10% HI-FBS. PBMC were isolated from the normal donor blood apheresis cones using a Ficoll gradient and activated overnight with 5 μg/mL of phytohemagglutinin (PHA-L; Millipore, cat#431784, Burlington, MA, USA). PBMC were added to each well at a ratio 1:10 and 1:20 (Target:Effector). Each condition was performed in triplicate. The calcein fluorescence was recorded every 10 min for 16 h using the EVOS FL Auto (Thermo) system equipped with the onstage incubator set at 37 °C and 5% CO_2_. The total number of calcein-positive cells and fluorescence intensities of intracellular calcein were calculated for each condition. The tumor cell viability was calculated at 5 h post-incubation. The survival index was obtained by the mean calcein fluorescence in treated cells divided by the mean fluorescence in the control (tumor cells only). An image analysis was performed using Fiji software (National Institute of Health, Bethesda, MD, USA).

### 2.7. Statistical Analysis

The normality of distribution was assessed using the Shapiro-Wilk normality test. The Student’s *t*-test (parametric) and Mann–Whitney *U* test (nonparametric) were employed to compare the two groups. A paired Wilcoxon test (Wilcoxon’s signed rank test) was performed to analyze the data presented in Figure 6C. The results were considered significant for *p*-values < 0.05. The *p*-values were either specified in the figure or denoted as asterisk: * *p* < 0.05, ** *p* < 0.01, and *** *p* < 0.001. All data were analyzed and plotted in GraphPad Prism 9.0.1 (San Diego, CA, USA).

Additional materials and methods for the Western blot, immunohistochemistry, and flow cytometry can be found in the Supplementary Materials.

## 3. Results

### 3.1. Identification of Cell Surface Protein Repertoires in RMS and Normal Muscle

**Figure 1:**
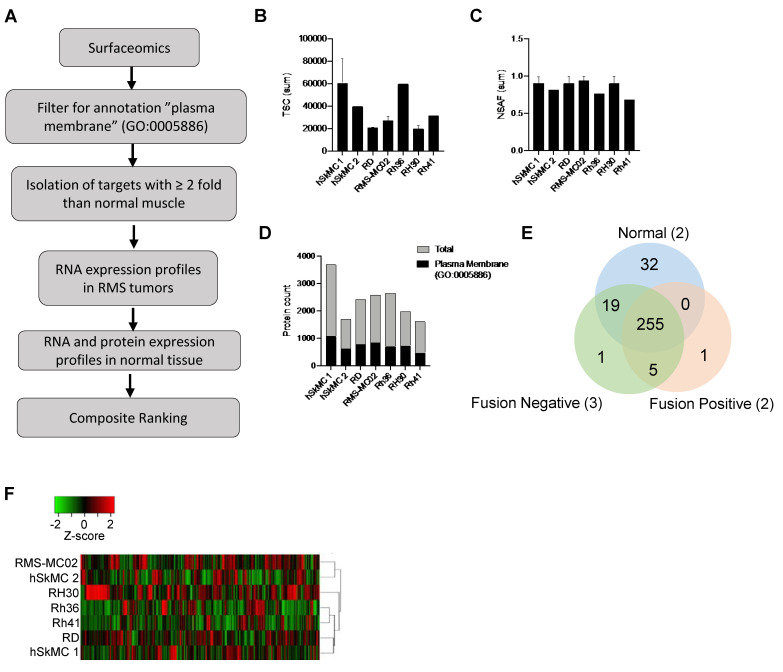
Distinct cell-surface protein signatures are identified in RMS subtypes and normal muscle. (**A**) Overall representation of the bioinformatics strategy to identify highly confident RMS-enriched cell surface proteins by combined proteomic and transcriptomic analysis of RMS. (**B**) Sum of TSC (total spectral count) and (**C**) NSAF (normalized spectral abundance factor) for each cell line analyzed. (**D**) Total protein count for each cell line. Count of proteins with the annotation plasma membrane is superimposed on the total protein count (black bars). (**E**) Venn diagram showing number of proteins identified in all cell lines of each subtype. (**F**) Heatmap of hierarchical clustering for cell surface protein data set filtered with the annotation “plasma membrane” (GO:0005886), Average linkage, Euclidean distance.

In order to define a set of cell surface proteins with an extracellular domain that can serve as potential therapeutic targets in RMS, we used a combination of cell surface capture and proteomic profiling on five RMS cell lines (three fusion-negative and two fusion-positive) and two normal skeletal muscle cell lines (Figure 1A). The proteins expressed in all fusion-negative and/or fusion-positive RMS cell lines by more than two-fold with a false discovery rate <0.05 compared to normal muscle were selected as target candidates. While the biotinylation method enriches for cell surface proteins, mass spectrometry can still reveal the cytosolic proteins interacting with plasma membrane proteins that are considered as “false” positives. To validate the subcellular location of the proteins identified, several publicly available databases were interrogated (Figure S1A). Gene Ontology GO:0005886 encompassed other annotation databases by providing the most exhaustive list of cell surface proteins. By using GO:0005886 for filtering data, we ensured not losing the true positives. An independent transcriptomic dataset (GSE108022) of 101 RMS (66 fusion-negative and 35 fusion-positive) and five normal skeletal muscle tissues was used to determine the expression profiles of the genes coding for cell surface proteins identified by mass spectrometry [5]. The publicly transcriptomic dataset Genotype-Tissue Expression (GTEx) containing gene expression profiles of 54 different human tissues from 948 donors was used to determine the expression of the target candidates in normal tissues. Finally, we also included the expression of target proteins obtained from a recent large-scale proteomic profiling of normal organs [19]. RNA and protein expression profiles in RMS and normal tissue were used to create a composite rank and identify targetable RMS-specific/enriched cell surface proteins.

Upon an analysis of the cell surface proteome by label-free mass spectrometry, significant differences in the number of peptides (total spectral counts, TSC) were detected between individual samples (Figure 1B). To normalize our data, we employed a normalized spectral count by the protein length (NSAF), which improves the quantification of protein abundance with label-free proteomics. Upon applying NSAF, the total spectral counts were normalized, and no statistically significant differences the between samples were observed (Figure 1C). A total of 5061 different proteins were found in combined cell lines (Figure 1D). Protein identification revealed an average of 2676 proteins detected per cell line, with hSkMC1 showing the highest number of detected proteins (3694 proteins) (Table S1). From the pool of proteins identified by mass spectrometry, an average of 1455 proteins were located at the plasma membrane (Table S2). When the filtering step was applied with Gene Ontology GO:0005886 to tease out the proteins with incorrect subcellular locations, we found an average of 31.6% of the proteins annotated as located at the plasma membrane. The protein repertoires were heterogeneous within types of cell lines (Figure S1B–D and Figure 1D). Despite sharing similarities, several proteins were found exclusively in one cell line compared to the other cell lines of the same group. When diseased and normal muscle-specific protein repertoires were compared, we identified a total of 255 proteins commonly expressed by the two normal skeletal muscle cell lines and the five RMS cell lines (Figure 1E). We found seven and 32 cell surface proteins specifically expressed by RMS cells and normal muscle cells, respectively. When the RMS cell lines were grouped based on their fusion status, only one protein was exclusively expressed in each RMS subtype. The unsupervised hierarchical clustering of the cell lines based on the protein repertoires showed four clusters (Figure 1F). The fusion-negative RMS cell lines RMS-MC02 and hSkMC1 segregated together, while the fusion-negative RMS cell lines RD and hSkMC2 clustered together. Fusion-positive RH41 and fusion-negative RH36 segregated together, and fusion-positive RH30 was separated from all other cell lines. The proteomic profiling of the RMS cell lines and normal skeletal muscle revealed cell type-specific surfaceome signatures, but it did not differentiate the RMS from normal muscle and fusion-positive from fusion-negative RMS cells.

### 3.2. Validation of RMS-Enriched Cell Surface Proteins by Combined Proteomic and Transcriptomic Analysis

**Figure 2:**
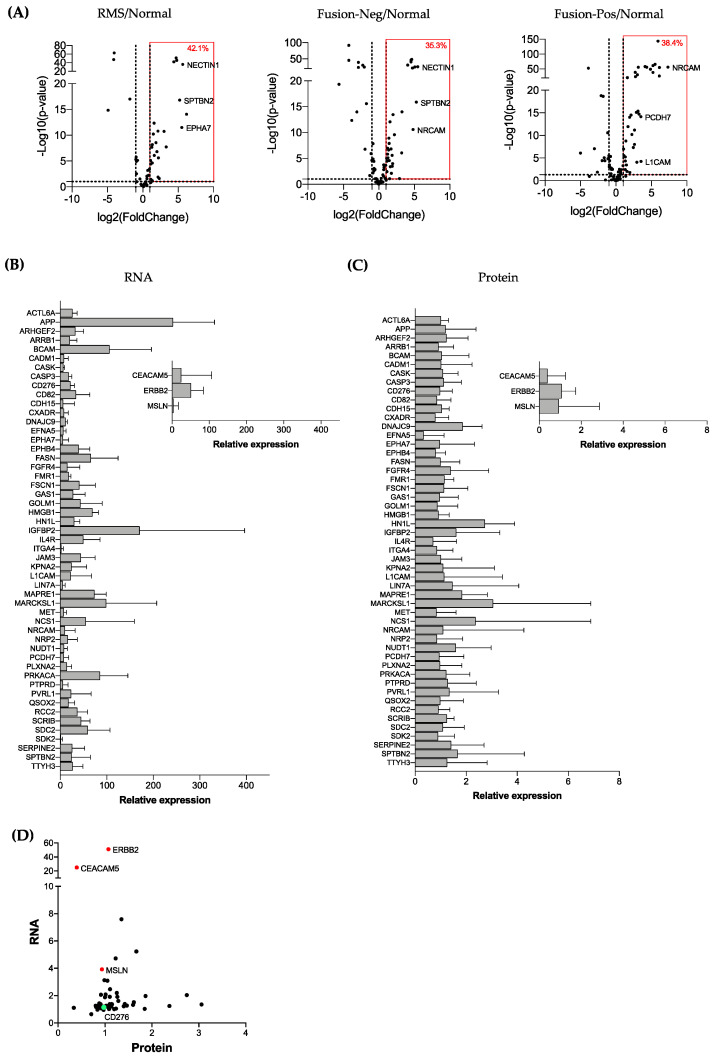
Validation of RMS-enriched cell-surface proteins by combined proteomic and transcriptomic analyses. (**A**) Volcano plot reporting *p*-values against fold changes for transcriptomic analysis of RMS tumors and normal muscle. The red box represents significantly up-regulated genes. Relative expression of (**B**) RNA and (**C**) protein of RMS-enriched cell-surface antigens in normal tissues including three CAR-T targets. (**D**) Correlation analysis between RNA and protein relative expression of RMS-enriched cell-surface antigens in normal tissues. Selected CAR-T targets and CD276 (B7-H3) are represented in red and green, respectively.

The abundance of the cell surface proteins was compared between the normal muscle, fusion-negative, and fusion-positive RMS cell lines. A high degree of correlation in the protein expression was observed for the two normal skeletal muscle cell lines (Pearson’s *r* = 0.7994 *p* < 0.0001). Among the fusion-negative and fusion-positive cell lines, positive associations were also observed, but the correlation coefficients varied from 0.2829 (fusion-positive) to 0.4736 (fusion-negative) (Figure S1E). Among the cell surface proteins commonly expressed in fusion-negative and fusion-positive cell lines, 57 proteins were upregulated by more than two-fold in RMS compared to normal muscle (Table S3). Similarly, 85 and 99 proteins were upregulated by more than two-fold in fusion-negative and fusion-positive RMS, respectively (Tables S4 and S5). To identify high-confidence cell surface targets in RMS, we interrogated a transcriptomic dataset (GSE108022) that consists of 66 fusion-negative RMS tumors, 35 fusion-positive RMS tumors, and 5 normal muscle tissues to analyze the gene expressions of our target candidates [5]. By using a two-fold increase and a *p*-value of 0.05 as the cut-off, we found that 42.1% (24 out of 57) of the targets overexpressed in all RMS cell lines at the protein level were also increased at the transcriptional level (Figure 2A and Table S6). When the samples were sub-grouped based on their fusion status, 35.3% (30 out of 85) and 38.4% (38 out of 99) of the targets were upregulated in RMS compared to normal muscle at the RNA level (Tables S7 and S8).

One major barrier to the development of effective antibody-based therapies is the expression of tumor targets within normal tissue causing on-target, off-tumor toxicity [21]. To improve the safety and minimize the off-tumor toxicity, tumor-enriched cell surface proteins must have a limited expression in normal organs. To determine which RMS-enriched cell surface proteins may be suitable as targets for antibody-based therapies, we analyzed their expression in normal tissues using publicly available GTEx transcriptomic and proteomic datasets [19,22] (Figure 2B–D). We also included Mesothelin (MSLN), CEACAM5, and HER2 (ERBB2), three common targets for CAR-T therapy in solid tumors demonstrating an acceptable safety profile in humans [23,24]. Among the three selected CAR-T targets, ERBB2 was the most expressed in normal tissue at the RNA level, followed by CEACAM5 and MSLN (Figure 2B and Table S9). Many (80.3%) (41/51) of the RMS-enriched cell surface proteins had lower normal gene expression than ERBB2. The proteomic data showed that ERBB2 was also the most expressed protein among the three CAR-T targets (Figure 2C and Table S10), and 49% (25/51) of the RMS-enriched cell surface proteins had a lower relative protein expression compared to ERBB2. The least and most abundant proteins in normal tissue were EFNA5 and MARCKSL1, respectively. A correlation analysis of the protein and RNA relative expression in normal tissues showed that most RMS-associated surface antigen candidates have a lower RNA expression and similar protein expression than the three CAR-T targets (Figure 2D).

Based on the RNA and protein expression on the tumor and normal tissues of cell surface antigens, we generated a composite rank by assigning equal weights to transcriptomic and proteomic ranks of overexpressed genes and proteins in RMS (Table 1, Table 2 and Table 3). For tumor expression, the gene and protein with the highest expression was assigned rank 1. For normal expression, the gene and protein with the lowest expression was assigned rank 1. A gene enrichment analysis revealed the enrichment of the proteins associated to neurogenesis, axon guidance, and cell adhesion (Figure S2).

Using combined proteomic and transcriptomic analyses of RMS tumors and normal tissue, we identified several cell surface proteins enriched in RMS and with limited expression in normal tissue, suggesting they are potential RMS target candidates for antibody-based therapies.

### 3.3. The Immune Checkpoint Molecule B7-H3 Is Upregulated in Both Fusion-Negative and Fusion-Positive RMS

Among the cell surface proteins that were upregulated at the RNA and protein levels in RMS compared to the normal muscle and limited expression in normal tissue, we identified the immune checkpoint molecule B7-H3 (Table 1, Table 2 and Table 3 and Figure 2D). B7-H3 (CD276) is a member of the B7 family that consists of 10 immune checkpoint molecules, including the well-known immunotherapeutic target B7-H1 (PD-L1) [25]. B7-H3 represents a promising target for the treatment of RMS, because it not only appears as one of the most upregulated cell surface antigens in tumors but, also, its immunoregulatory function may provide novel insights on the mechanisms associated with tumor immune evasion for the development of new immune checkpoint inhibitors.

**Figure 3:**
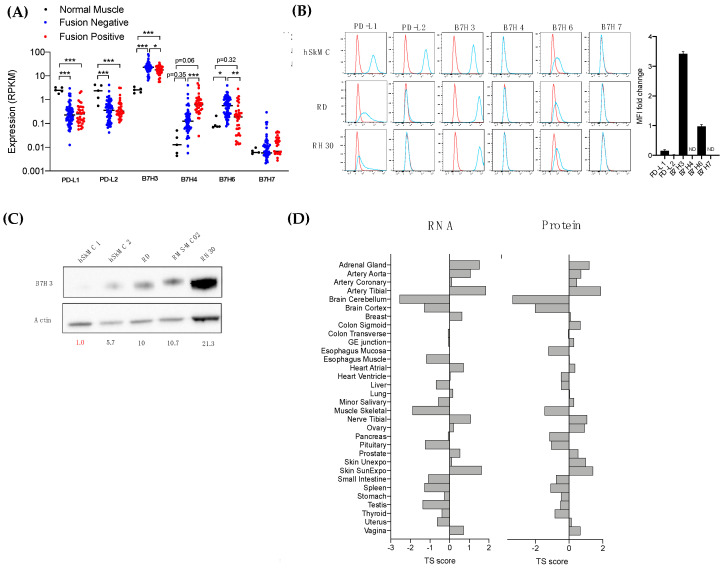
Expression of the immune checkpoint molecule B7-H3 in RMS and normal tissue. (**A**) Normalized expression (RPKM) of B7 molecules from RNA sequencing of normal muscle (N = 5), fusion negative (N = 66), and fusion positive (N = 35) RMS samples. Each data point represents an individual sample. The median is noted by a horizontal bar and significance denoted by asterisks (*** *p* < 0.001; ** *p* < 0.01; * *p* < 0.05; n.s., not significant, Student’s *t*-test). (**B**) Histograms of flow cytometric analysis of B7 molecule expression in RMS and normal muscle cell lines. Bar graph shows mean fluorescence intensity (MFI) fold change in RMS cell lines compared to normal muscle. ND indicates “not detectable” expression (**C**) Western blot of B7H3 expression in 5 different cell lines. Band intensity for B7-H3 normalized on actin is indicated for each cell line. (**D**) Tissue Specific (TS) score of protein and RNA expression of B7H3 in each normal tissue (obtained from Jiang et al. [15].

The Gene expression profiles of the B7 molecules showed B7-H3 as the most expressed B7 molecule in RMS, and the tumor expression was significantly higher in 100% of both fusion-negative RMS (9.45-fold) and fusion-positive RMS (7.03-fold) (Figure 3A). The B7-H6 gene expression was significantly increased in fusion-negative RMS. B7-H4 was upregulated in both RMS subtypes but at a greater extent in fusion-positive RMS (*p* = 0.06). Strikingly, both PD-L1 (B7-H1) and PD-L2 (B7-DC) were significantly downregulated in RMS compared to normal muscle. The cell surface protein expression of the B7 molecules was also assessed by flow cytometry in normal skeletal muscle cells, fusion-negative (RD), and fusion-positive RMS (RH30) (Figure 3B). Similar findings were obtained with B7-H3 being the most expressed B7 member, with a 3.42-fold increase in RMS cells. In contrast, PD-L1 and PD-L2 were downregulated in RMS, which corroborates with transcriptomic data. While B7-H4 and B7-H7 were not detected on the surfaces of normal and cancer cells, B7-H6 were weakly expressed in normal muscle and downregulated in RMS cell lines. In the proteomic data, the B7-H6 protein was expressed at low levels in three RMS cell lines (Table S1). A Western blot of the cell lines showed a similar increase of the total B7-H3 proteins in the RMS cell lines compared to the normal muscle (Figure 3C). We analyzed the tissue specificity score of B7-H3 in normal tissue as described previously [19]. The tissue specificity (TS) score defines the enrichment of B7H3 across normal tissues. A score superior of 2.5 means a protein is tissue-enriched, and a score superior of 4 defines a tissue-specific protein. An expression analysis in normal tissue showed a positive correlation between the RNA and protein expression of B7-H3 (Spearman correlation *ρ* = 0.84), and no tissue specificity was observed (Figure 3D).

To validate the differential expression of B7-H3 in normal tissue and RMS tumors, we first evaluated the sensitivity and specificity of four commercial antibodies using tumor xenografts of RMS wild-type and knockout cells for B7-H3 (Figure S3). While all antibodies showed positive signals and well-defined membrane staining in RH30 wild-type cells, no unspecific signal was detected with the AF1027 and EPNCIR122 antibodies (Figure S3C). The AF1027 clone was selected for tissue staining.

**Figure 4:**
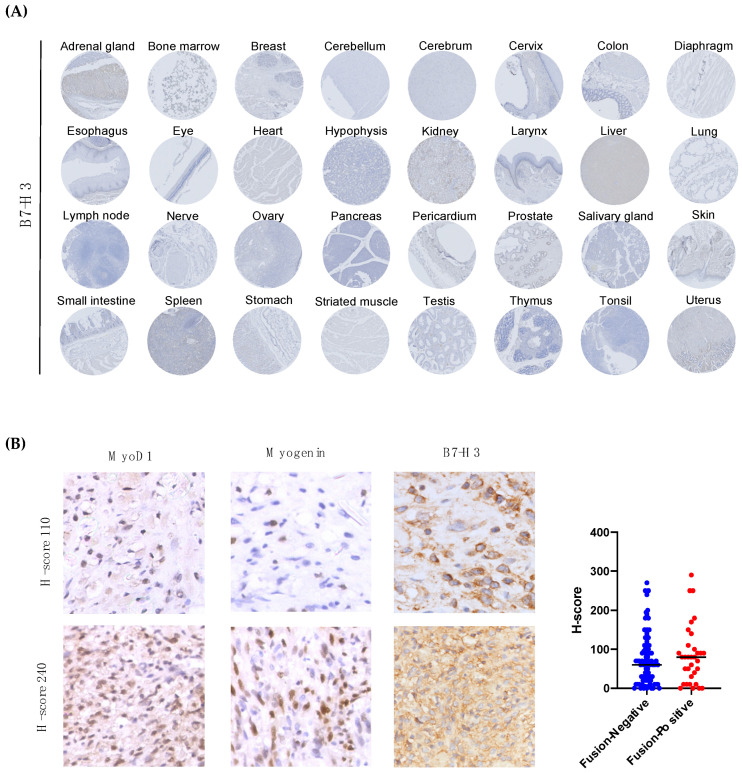
B7-H3 tissue expression in normal organs and RMS tumor specimens. (**A**) Immunohistochemical staining of B7H3 in a microarray of 32 normal tissues. Representative picture of triplicates for each tissue. (**B**) Representative picture of immunohistochemistry for MyoD1, Myogenin and B7H3 in RMS tumors. Scatter plot shows quantification of B7-H3 expression using H-scoring system in fusion-negative and fusion-positive RMS tumors (N = 132).

We performed staining on a normal human tissue microarray and RMS tumor sections. The tissue microarray consisted of three specimens for 32 normal tissues. No positive detection of B7-H3 was observed in normal human tissues (Figure 4A). We also analyzed B7-H3 tissue expression in a cohort of 132 RMS specimens (*N* = 97 patients) obtained from our institutional tissue registry (Table S11). B7-H3 expression was detected in 122/132 specimens (91.5%) with only 10 samples (8.5%) with negative expression. The RMS tumor specimens showed various levels of B7-H3 expression and intratumoral heterogeneity (Figure 4B). The staining of RMS tumor cells with Myogenin and MyoD1 revealed that B7-H3 is expressed by tumor cells and not detected in the stroma or tumor-infiltrating immune cells. The quantification of B7-H3 expression did not show any statistical differences between FN-RMS and FP-RMS. The median H-score was 60 and 80 for FN-RMS and FP-RMS, respectively. Furthermore, no association was observed between the B7-H3 expression and histological or molecular subtype (Table S11). Altogether, these findings showed that B7-H3 is not expressed by normal tissue at both the RNA and protein levels, while it is strongly expressed in most RMS tumors, regardless of the molecular subtype. 

### 3.4. Tumor B7-H3 Overexpression Is Associated with Low Infiltration of CD8^+^-T Cells in RMS Tumors and Impaired Antitumor Immune Response

**Figure 5:**
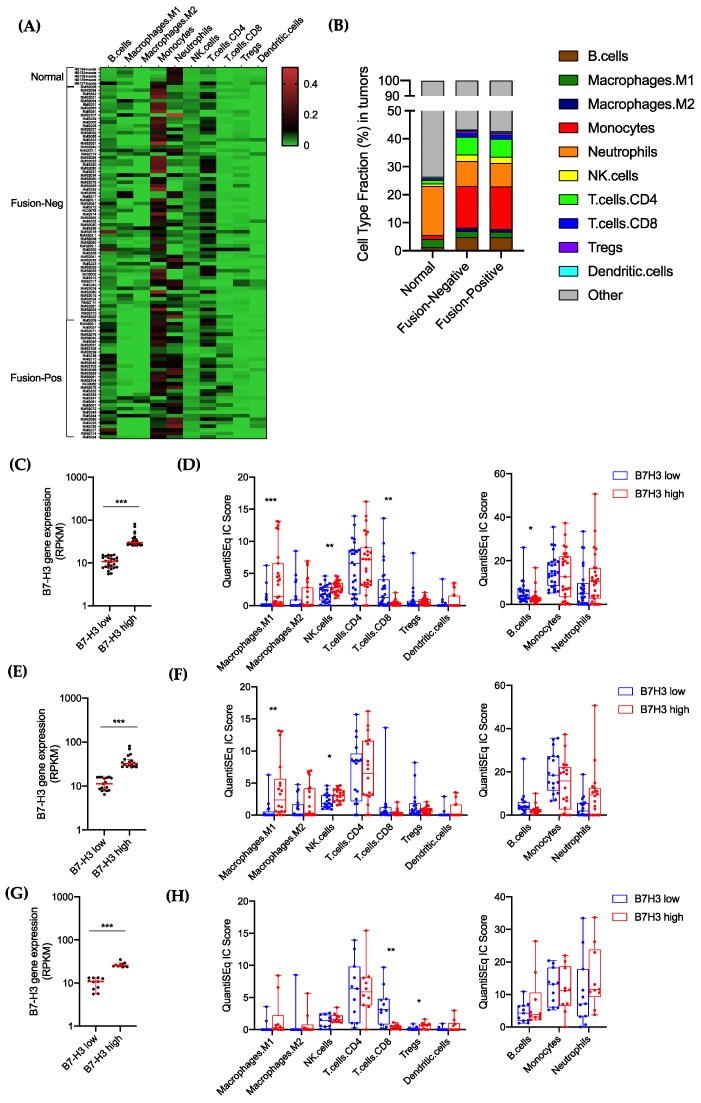
Relationship of B7-H3 expression and tumor-infiltrating immune cells in RMS. (**A**) Heatmap of immune cell abundance estimated by QuantiSeq analysis in normal muscle, fusion-negative and fusion-positive RMS. (**B**) Mean percentages of different immune cell subsets in normal muscle, fusion-negative and fusion-positive RMS. (**C**,**E**,**G**) B7-H3 RNA expression in quartile subgroups (25% highest, 25% lowest) in RMS tumors (**C**), FN-RMS (**E**) and FP-RMS (**G**) used for immune cell abundance analysis. (**D**,**F**,**H**) Bar graphs showing immune cell abundance between B7-H3-low and B7-H3-rich RMS tumors (**D**), FN-RMS only (**F**) and FP-RMS only (**H**). Significance is denoted by asterisks (*** *p* < 0.001; ** *p* < 0.01; * *p* < 0.05; Student’s *t*-test).

B7-H3 has shown paradoxical roles in tumor immunity [26]. Described originally as a costimulatory molecule, B7-H3 has also been linked to inhibition of the antitumor immune response and immune evasion [27,28]. The immunomodulatory role of B7-H3 and its relationship with tumor-infiltrating immune cells in RMS remains unknown. To address this unmet need, we investigated the immune landscape of fusion-positive and fusion-negative RMS by a deconvolution analysis of bulk RNA-seq data. We employed QuantiSeq, a computational pipeline, for the characterization and quantification of 10 tumor-infiltrating immune cell subsets [20]. An additional population called “Other” includes nonimmune cells such as malignant cells and fibroblasts. The relative abundance of different immune cell subsets was determined for normal muscle, fusion-negative, and fusion-positive RMS (Figure 5A,B). Higher proportions of monocytes, B-cells, macrophages M2, NK cells, CD4-T cells, CD8-T cells, and regulatory T cells were observed in both fusion-negative and fusion-positive RMS compared to normal muscle. In contrast, a higher content in neutrophils was found in normal muscle. Monocytes, neutrophils, and CD4-T cells represented ~70% of the immune cell content in RMS tumors, and no difference was observed between fusion-negative and fusion-positive RMS.

To determine the relationship between B7-H3 expression and tumor-infiltrating immune cells, we measured the immune cell abundance in B7-H3-low and B7-H3-high tumors using quartile subgroups (25% highest and 25% lowest). Regardless of the fusion status, B7-H3-high tumors were enriched in M1 macrophages and NK cells and depleted in CD8-T cells and B cells (Figure 5C,D). In fusion-negative RMS only, M1 macrophages and NK cells were significantly higher in B7-H3-rich tumors. Despite a trend toward a decrease of CD8-T-cell abundance in B7-H3-rich tumors, it was not statistically significant (Figure 5E,F). In fusion-positive RMS, only the infiltration of regulatory T cells and CD8-T cells were significantly affected by B7-H3 expression, where B7-H3-rich tumors have a higher infiltration of Tregs and depletion of CD8-T cells (Figure 5G,H). Correlation matrices were used to determine the degree of relationships between B7-H3 expression and the abundance of different immune cell subsets (Table S12). In fusion-negative RMS, a significant positive correlation was found between B7-H3 and M1 macrophages and neutrophils. A moderate negative correlation was also observed between B7-H3 and M2 macrophages and monocytes. Only CD8-T cells and B7-H3 showed a significant negative correlation in both fusion-negative and fusion-positive RMS.

**Figure 6:**
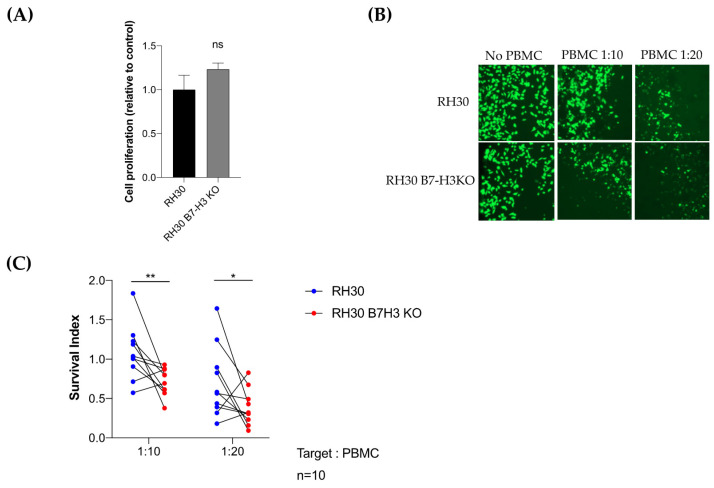
Loss of B7-H3 expression in RMS tumor cells is associated with higher T-cell cytotoxicity. (**A**) Histogram showing relative proliferation of B7-H3 knockout RH30 cells compared to wild-type cells. (**B**) Representative pictures of calcein-labeled wild-type and B7-H3 knockout RH30 cells after co-incubation with activated PBMCs for 4 hours. (**C**) Survival index of wild-type and B7-H3 knockout RH30 cells co-cultured with activated PBMC (N = 10 normal donors) at a ratio target: PBMC of 1:10 or 1:20. (** *p* < 0.01; * *p* < 0.05, Paired *t*-test).

To confirm a potential association between B7-H3 and CD8-T cells, we performed a T-cell cytotoxicity assay by coculturing activated PBMC isolated from 10 healthy donors with RH30 wild-type or knockout cells for B7-H3 and monitored the tumor cell survival over time. No significant difference was observed in B7-H3 knockout RH30 cells compared to the wild type (Figure 6A). For eight out of 10 normal donors, the loss of B7-H3 was associated with an increase in tumor cell killing (Figure 6B,C). A decrease of 34% and 45% in tumor cell survival was observed with B7-H3 knockout tumor cells at a ratio of 1:10 and 1:20 RH30:PBMC, respectively.

Altogether, these data show that B7-H3 tumor expression is associated with the distinct immune composition of RMS tumors rich in M1 macrophages, M2 macrophages, and neutrophils and depleted in T cells. In addition, B7-H3 expression in RMS is associated with the inhibition of T-cell cytotoxic functions.

## 4. Discussion

In this study, we conducted the first comprehensive characterization of the cell surface proteome (surfaceome) of RMS tumors using cell surface capture and mass spectrometry-based proteomics to identify new therapeutic targets. As a result of the technological advances and reduced costs, conventional proteomics has become an attractive tool for target discovery and therapeutic development. However, it suffers from low sensitivity for cell surface proteins, which are significantly less abundant and soluble than cytosolic proteins [29]. Although surfaceomic approaches have been shown to circumvent this challenge and enhance target discovery, it has not been widely adopted, as several challenges have limited its use [30,31,32]. It requires a large number of starting living cells that may complicate the use of primary cells that have a limited number of passages in 2D cultures. Alongside the established RMS cell lines, we successfully analyzed the surfaceome of one primary cell line cultured from a patient-derived RMS (RMS-MC02), while three other primary cell lines did not reach the number of cells desired. Although a limited number of cell lines have been used for surfaceomic profiling, we included a bioinformatic analysis of publicly available transcriptomic and proteomic datasets of RMS specimens and normal tissues to reveal high-confidence therapeutic targets for the treatment of RMS. We benefited from the RNA sequencing and proteomic profiling of normal organs to determine the basal expression of RMS-enriched surface proteins in human organs. The success of antibody-based and cell-based therapies relies not only on the recognition of an antigen highly expressed on tumor cells but, also, on the minimal on-target off-tumor toxicities caused by the expression of the same antigen on normal cells [23]. By analyzing the gene and protein expressions in RMS and normal tissue, we uncovered a repertoire of surface antigens targetable with targeted therapies and immunotherapies.

Among the cell surface proteins enriched in FN-RMS and FP-RMS, we rediscovered several molecules previously reported by other groups [5]. For instance, we identified the FGFR4 receptor overexpressed in FN-RMS, which is in line with the prior observations of FGFR4 mutation and amplification in this RMS subtype [5]. Several well-known *PAX3/7-FOXO1* target genes were also reported among the top cell surface proteins in FP-RMS, including MET, IL4R, FMR1, and NRCAM [33]. Interestingly, FMR and NRCAM were also upregulated in FN-RMS, suggesting similarities in the protein repertoires regardless of the fusion status. An outstanding work from Shern et al. showed that fusion-negative and fusion-positive RMS display common altered pathways, including the RAS/PIK3CA axis [5]. The hierarchical clustering of RMS surfaceome signatures does not separate fusion-negative and fusion-positive RMS, which corroborates with the previous findings. Gene overexpression either through *PAX3/7-FOXO1* activity or mutation-associated amplification in FN-RMS may explain the high similarities in the cell surface protein repertoire of both molecular subtypes. This is of utmost interest, as it suggests that therapeutic strategies can be designed to target proteins commonly enriched in both RMS subtypes.

Our surfaceomic analysis also revealed new targetable RMS-enriched cell surface proteins. Among the commonly overexpressed proteins in both RMS subtypes, we identified the cell adhesion molecule CDH4 and ephrin receptors EFNA5 and EPHA7. CDH4 (R-Cadherin) has been previously found amplified in 43.6% of osteosarcomas, and its overexpression was associated with metastasis and a poor prognosis [34]. Similarly, CDH4 is an important driver of metastasis in glioblastoma [35]. In skeletal muscle, CDH4 has been reported to block myogenesis process and induce myoblast transformation, suggesting a potential oncogenic role in rhabdomyosarcoma [36]. Ephrin A5 and A7 are tyrosine kinase receptors that both bind the ephrin A5 ligand [37]. Ephrin receptors play a large role in embryonic and neural development [38]. In disease, Ephrin receptors and ligands can mediate the metastatic potential of cancer cells [39]. Little is known about the exact roles of Ephrin A5 and A7 in cancer progression. A few studies point towards a paradoxical role with pro- and antimetastatic functions, suggesting disease-specific activity [40,41,42]. The identification of new RMS-enriched tumor antigens, such as CDH4, Ephrin A5, and A7, support the clinical relevance of using surfaceomics for target discovery and drug development. Moreover, it provides novel biological insights into RMS pathology. Further studies are warranted to elucidate the roles of CDH4 and Ephrin A5/A7 in RMS, paving the way for the development of new treatments preventing disease recurrence and metastasis.

Another interesting target identified in our study is the immune checkpoint molecule B7-H3. B7-H3 is a member of the B7 family that contains ten members, including the well-known immune checkpoint PD-L1, the target of FDA-approved immunotherapies [25]. We identified B7-H3 as the major B7 immune molecule expressed on the surface of RMS cells. In our cohort of RMS specimens, only 8.55% of samples tested were negative for B7-H3, and no difference was observed between FN-RMS or FP-RMS. Interestingly, PD-L1 was poorly expressed in RMS cells, and it was significantly lower than in normal muscle. This is in accordance with the previous reports of a minimal or negative expression of PD-L1 in rhabdomyosarcoma [43,44]. B7-H3 has a multifaceted role in cancer, including immunological and nonimmunological functions [45,46]. Herein, we provided the first biological insights of the B7-H3 role in RMS. We analyzed the impact of B7-H3 expression in the immune composition of RMS tumors by deconvolution of the RNA-seq data. Interestingly, we found that RMS tumors rich in B7-H3 are depleted in CD8-T cells. Furthermore, B7-H3 knockout in RMS cells was associated with greater T-cell-mediated cytotoxicity. Altogether, this suggests that B7-H3 acts as an immune-inhibitory molecule in RMS. The underlying molecular and cellular mechanisms of B7-H3-mediated antitumor immunity remain to be elucidated, and unlike PD-L1, the B7-H3 receptor on immune cells has not been identified yet. Interestingly, we also found a positive correlation between B7-H3 expression and an abundance of neutrophils and M1 macrophages in FN-RMS. In colorectal cancer and hepatocellular carcinoma, B7-H3 tumor expression was associated with a higher infiltration of CD68^+^ macrophages and the polarization of M1 to M2 macrophages [47,48]. While it was not statistically significant, we also observed higher infiltration of M2 macrophages in B7-H3-rich FN-tumors. This suggests that B7-H3 may drive the polarization of M1 macrophages towards the tumor-promoting M2 phenotype. Finally, the overexpression of B7-H3 in tumors compared to normal muscle was associated with a higher monocyte infiltration. Tumor-associated monocytes can differentiate into myeloid suppressor-derived cells, which are an important contributor of the immunosuppressive tumor microenvironment. While we cannot exclude a role of B7-H3 in MDSC differentiation, B7-H3 can also be expressed by MDSCs and contribute to CD8-T-cell inhibition and tumor progression [49,50,51,52].

While clinical trials evaluating PD-L1/PD-1 inhibitors in pediatric sarcoma have been unsuccessful, B7-H3 has become a popular target for new, targeted therapies [53]. Antibody–drug conjugates and CAR-T therapy are currently evaluated in clinical trials and are poised to positively change the therapeutic landscape of childhood cancers [54,55,56]. Our work provides novel mechanistic insights into the role of B7-H3 in tumor immune evasion and RMS progression. A complete characterization of B7-H3 function and regulation will pave the way for developing new B7-H3-based immunotherapies for the treatment of RMS.

## 5. Conclusions

While clinical trials evaluating PD-L1/PD-1 inhibitors in pediatric sarcoma have been unsuccessful, B7-H3 has become a popular target for new, targeted therapies [49]. Antibody–drug conjugates and CAR-T therapy are currently being evaluated in clinical trials and are poised to positively change the therapeutic landscape of childhood cancers [50,51,52]. Our work provides novel mechanistic insights on the role of B7-H3 in tumor immune evasion and RMS progression. A complete characterization of the B7-H3 functions and regulations will pave the way for developing new B7-H3-based immunotherapies for the treatment of RMS.

## Figures and Tables

**Table 1:** Composite ranking of RMS-enriched cell-surface proteins based on combined transcriptomic and proteomic analyses.

Entry	Gene Names	Gene	Proteomic RMS	Transcriptomic RMS	Proteomic Normal	Transcriptomic Normal	Composite Sum	Composite Rank
P55283	CDH4	CDH4	1	12	1	1	15	1
P52803	EFNA5 EPLG7 LERK7	EFNA5	1	9	2	4	16	2
Q92823	NRCAM KIAA0343	NRCAM	4	1	13	6	24	3
O60245	PCDH7 BHPCDH	PCDH7	9	8	5	5	27	4
Q15375	EPHA7 EHK3 HEK11	EPHA7	18	3	7	2	30	5
Q15223	NECTIN1 HVEC PRR1 PVRL1	NECTIN1	1	2	19	14	36	6
O14936	CASK LIN2	CASK	5	21	12	3	41	7
O60462	NRP2 VEGF165R2	NRP2	7	22	3	9	41	7
P54826	GAS1	GAS1	8	14	6	17	45	9
Q06787	FMR1	FMR1	6	15	16	11	48	10
Q16658	FSCN1 FAN1 HSN SNL	FSCN1	12	5	14	19	50	11
Q6ZRP7	QSOX2 QSCN6L1 SOXN	QSOX2	20	11	10	10	51	12
Q9C0H2	TTYH3 KIAA1691	TTYH3	10	7	18	16	51	12
O15020	SPTBN2 KIAA0302 SCA5	SPTBN2	14	4	20	15	53	14
P32004	L1CAM CAML1 MIC5	L1CAM	15	10	15	13	53	14
Q5ZPR3	CD276 B7H3 PSEC0249 UNQ309/PRO352	CD276	23	13	8	12	56	16
O75051	PLXNA2 KIAA0463 OCT PLXN2 UNQ209/PRO235	PLXNA2	24	16	9	8	57	17
P09429	HMGB1 HMG1	HMGB1	11	24	4	22	61	18
Q8WXX5	DNAJC9	DNAJC9	17	18	22	7	64	19
P49006	MARCKSL1 MLP MRP	MARCKSL1	16	6	24	24	70	20
Q9H910	JPT2 C16orf34 HN1L L11	HN1L	13	19	23	18	73	21
P34741	SDC2 HSPG1	SDC2	22	20	11	21	74	22
Q15691	MAPRE1	MAPRE1	19	17	21	23	80	23
Q14160	SCRIB CRIB1 KIAA0147 LAP4 SCRB1 VARTUL	SCRIB	21	23	17	20	81	24

**Table 2:** Composite ranking of fusion-negative RMS-enriched cell-surface proteins based on combined transcriptomic and proteomic analyses.

Entry	Gene Names	Gene	Surfaceomic RMS	Transcriptomic RMS	Proteomic Normal	Transcriptomic Normal	Composite Sum	Composite Rank
P52803	EFNA5 EPLG7 LERK7	EFNA5	1	10	1	3	15	1
Q92823	NRCAM KIAA0343	NRCAM	8	4	12	7	31	2
Q15223	NECTIN1 HVEC PRR1 PVRL1	NECTIN1	1	1	19	13	34	3
O60245	PCDH7 BHPCDH	PCDH7	15	11	5	4	35	4
Q9BY67	CADM1 IGSF4 IGSF4A NECL2 SYNCAM TSLC1	CADM1	1	19	9	6	35	4
P27701	CD82 KAI1 SAR2 ST6 TSPAN27	CD82	1	14	2	20	37	6
O14910	LIN7A MALS1 VELI1	LIN7A	1	17	22	1	41	7
P22455	FGFR4 JTK2 TKF	FGFR4	13	3	20	9	45	8
O14936	CASK LIN2	CASK	7	26	11	2	46	9
Q8NBJ4	GOLM1 C9orf155 GOLPH2 PSEC0242	GOLM1	10	13	3	22	48	10
P54826	GAS1	GAS1	12	16	6	18	52	11
Q06787	FMR1	FMR1	9	18	15	12	54	12
Q9C0H2	TTYH3 KIAA1691	TTYH3	16	7	18	17	58	13
O15020	SPTBN2 KIAA0302 SCA5	SPTBN2	18	2	25	14	59	14
P18065	IGFBP2 BP2 IBP2	IGFBP2	1	5	24	29	59	14
P36639	NUDT1 MTH1	NUDT1	25	12	23	5	65	16
Q16658	FSCN1 FAN1 HSN SNL	FSCN1	24	8	14	21	67	17
Q6ZRP7	QSOX2 QSCN6L1 SOXN	QSOX2	26	24	7	10	67	17
P42574	CASP3 CPP32	CASP3	14	30	13	11	68	19
O96019	ACTL6A BAF53 BAF53A INO80K	ACTL6A	30	23	8	16	77	20
P07093	SERPINE2 PI7 PN1	SERPINE2	27	15	21	15	78	21
P09429	HMGB1 HMG1	HMGB1	20	28	4	26	78	21
P62166	NCS1 FLUP FREQ	NCS1	17	9	28	24	78	21
Q8WXX5	DNAJC9	DNAJC9	21	22	27	8	78	21
P34741	SDC2 HSPG1	SDC2	28	21	10	25	84	25
Q14160	SCRIB CRIB1 KIAA0147 LAP4 SCRB1 VARTUL	SCRIB	19	25	17	23	84	26
P05067	APP A4 AD1	APP	11	29	16	30	86	27
P49006	MARCKSL1 MLP MRP	MARCKSL1	23	6	30	28	87	28
Q9H910	JPT2 C16orf34 HN1L L11	HN1L	22	27	29	19	97	28
Q15691	MAPRE1	MAPRE1	29	20	26	27	102	30

**Table 3:** Composite ranking of fusion-positive RMS-enriched cell-surface proteins based on combined transcriptomic and proteomic analyses.

Entry	Gene Names	Gene	Surfaceomic RMS	Transcriptomic RMS	Proteomic Normal	Transcriptomic Normal	Composite Sum	Composite Rank
P55283	CDH4	CDH4	1	17	1	1	20	1
Q58EX2	SDK2 KIAA1514	SDK2	1	6	11	4	22	2
Q96KG7	MEGF10 KIAA1780	MEGF10	15	9	1	1	26	3
O00762	UBE2C UBCH10	UBE2C	23	2	1	1	27	4
P52803	EFNA5 EPLG7 LERK7	EFNA5	1	23	4	10	38	5
P49407	ARRB1 ARR1	ARRB1	1	13	13	18	45	6
P78310	CXADR CAR	CXADR	1	26	6	13	46	7
Q15375	EPHA7 EHK3 HEK11	EPHA7	22	3	17	6	48	8
Q92823	NRCAM KIAA0343	NRCAM	8	1	26	14	49	9
O60245	PCDH7 BHPCDH	PCDH7	13	12	15	11	51	10
P24394	IL4R IL4RA 582J2.1	IL4R	1	21	5	31	58	11
Q15223	NECTIN1 HVEC PRR1 PVRL1	NECTIN1	1	4	35	21	61	12
P23468	PTPRD	PTPRD	18	5	34	7	64	13
O60462	NRP2 VEGF165R2	NRP2	11	30	9	15	65	14
P08581	MET	MET	16	36	8	12	72	15
P13612	ITGA4 CD49D	ITGA4	38	22	10	5	75	16
O14936	CASK LIN2	CASK	9	33	25	9	76	17
P32004	L1CAM CAML1 MIC5	L1CAM	17	11	29	20	77	18
P52292	KPNA2 RCH1 SRP1	KPNA2	14	16	27	22	79	19
Q9P258	RCC2 KIAA1470 TD60	RCC2	26	14	12	27	79	19
Q06787	FMR1	FMR1	10	25	30	17	82	21
P54826	GAS1	GAS1	21	24	16	24	85	22
Q9C0H2	TTYH3 KIAA1691	TTYH3	19	10	33	23	85	22
Q6ZRP7	QSOX2 QSCN6L1 SOXN	QSOX2	36	15	19	16	86	24
P55291	CDH15 CDH14 CDH3	CDH15	37	19	23	8	87	25
Q92974	ARHGEF2 KIAA0651 LFP40	ARHGEF2	12	20	32	26	90	26
Q16658	FSCN1 FAN1 HSN SNL	FSCN1	28	7	28	29	92	27
Q5ZPR3	CD276 B7H3 PSEC0249 UNQ309/PRO352	CD276	29	27	18	19	93	28
P54760	EPHB4 HTK MYK1 TYRO11	EPHB4	31	32	7	28	98	29
Q9BX67	JAM3 UNQ859/PRO1868	JAM3	33	18	21	30	102	30
P09429	HMGB1 HMG1	HMGB1	20	38	14	34	106	31
P49006	MARCKSL1 MLP MRP	MARCKSL1	34	8	38	37	117	32
P50895	BCAM LU MSK19	BCAM	27	31	22	38	118	33
P49327	FASN FAS	FASN	32	34	20	33	119	34
Q9H910	JPT2 C16orf34 HN1L L11	HN1L	30	29	37	25	121	35
Q15691	MAPRE1	MAPRE1	25	28	36	35	124	36
P17612	PRKACA PKACA	PKACA	24	35	31	36	126	37
P34741	SDC2 HSPG1	SDC2	35	37	24	32	128	38

## Data Availability

All data presented here are available in the Supplementary Materials.

## Supplementary Materials

The following are available online at https://www.mdpi.com/article/10.3390/cancers13184528/s1, Table S1. Normalized protein abundance (expressed in NSAF values) in RMS and normal muscle cell lines; Table S2. List of proteins identified as cell-surface proteins (GO:00005886); Table S3. List of cell-surface proteins upregulated by 2-fold in RMS compared to normal muscle; Table S4. List of cell-surface proteins upregulated by 2-fold in fusion-negative RMS compared to normal muscle; Table S5. List of cell-surface proteins upregulated by 2-fold in fusion-positive RMS compared to normal muscle; Table S6. List of cell-surface proteins from Table S4 upregulated by 2-fold in RMS at the RNA level; Table S7. List of cell-surface proteins from Table S5 upregulated by 2-fold in fusion-negative RMS at the RNA level; Table S8. List of cell-surface proteins from Table S6 upregulated by 2-fold in fusion-positive RMS at the RNA level; Table S9. Normal tissue median RNA expression of RMS-enriched cell-surface proteins; Table S10. Normal tissue median protein expression of RMS-enriched cell-surface proteins; Table S11. B7-H3 tissue expression in RMS specimens and association with clinicopathological features; Table S12. Correlation analysis of B7-H3 expression with immune cell abundance in RMS tumors; Figure S1. Comparison of cell-surface protein repertoires in RMS and normal muscle; Figure S2. Gene enrichment analysis for RMS-enriched cell-surface proteins; Figure S3. Validation of antibody specificity for B7-H3 tissue staining.
