# Supplementary material for: Surfaceome Profiling of Rhabdomyosarcoma Reveals B7-H3 as a Mediator of Immune Evasion
Source: Cancers (Basel). 2021 Sep 9;13(18):4528. doi: 10.3390/cancers13184528 (PMC8466404; doi:10.3390/cancers13184528)
Supplement: Supplementary file 1 [file cancers-13-04528-s001.zip › Supplementary Figures.pptx]

## Slide 1
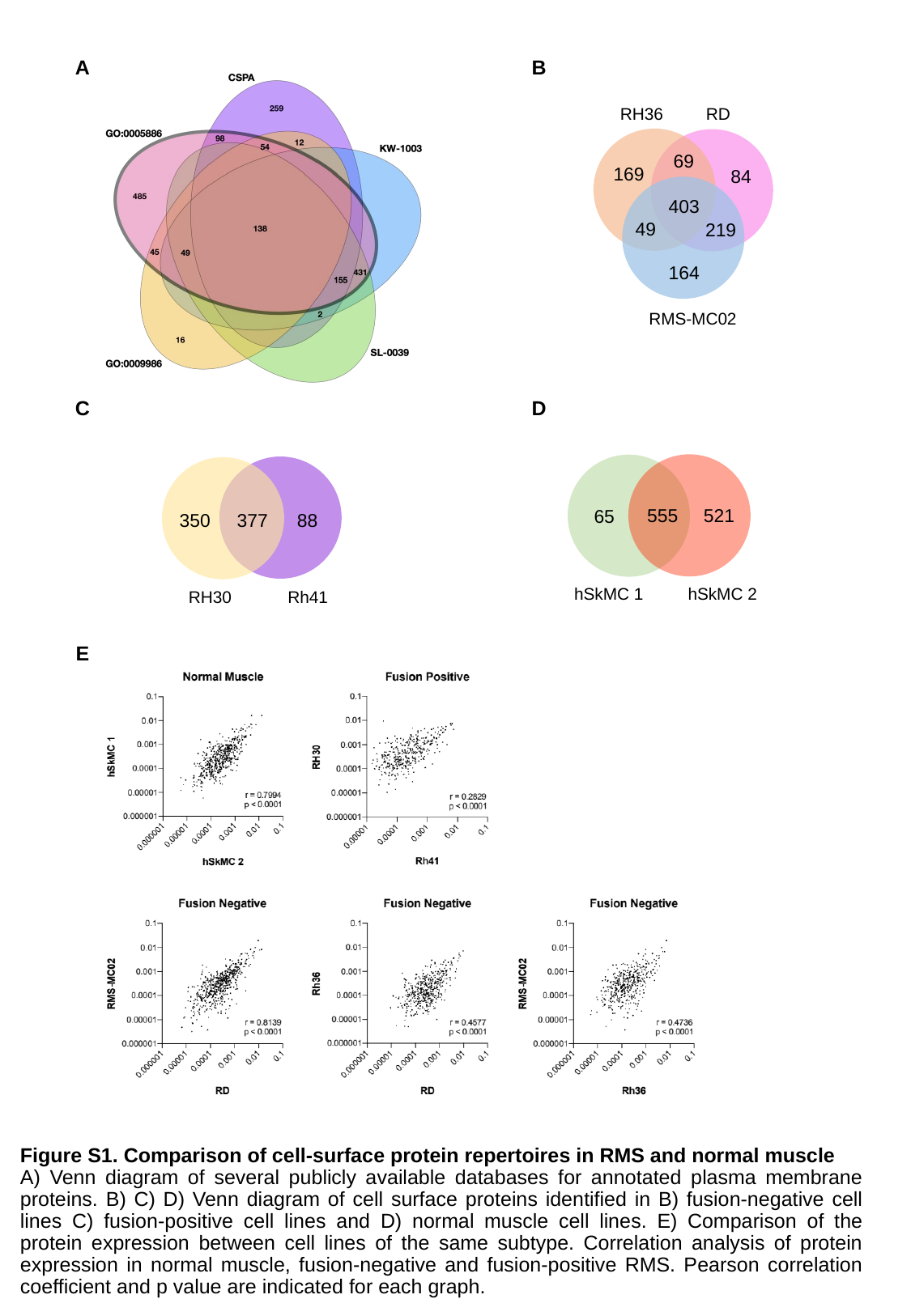

A
B
RD
RH36
69
169
84
403
49
219
164
RMS-MC02
C
D
555
521
65
hSkMC 2
hSkMC 1
350
377
88
Rh41
RH30
E
Figure S1. Comparison of cell-surface protein repertoires in RMS and normal muscle
A) Venn diagram of several publicly available databases for annotated plasma membrane proteins. B) C) D) Venn diagram of cell surface proteins identified in B) fusion-negative cell lines C) fusion-positive cell lines and D) normal muscle cell lines. E) Comparison of the protein expression between cell lines of the same subtype. Correlation analysis of protein expression in normal muscle, fusion-negative and fusion-positive RMS. Pearson correlation coefficient and p value are indicated for each graph.

## Slide 2
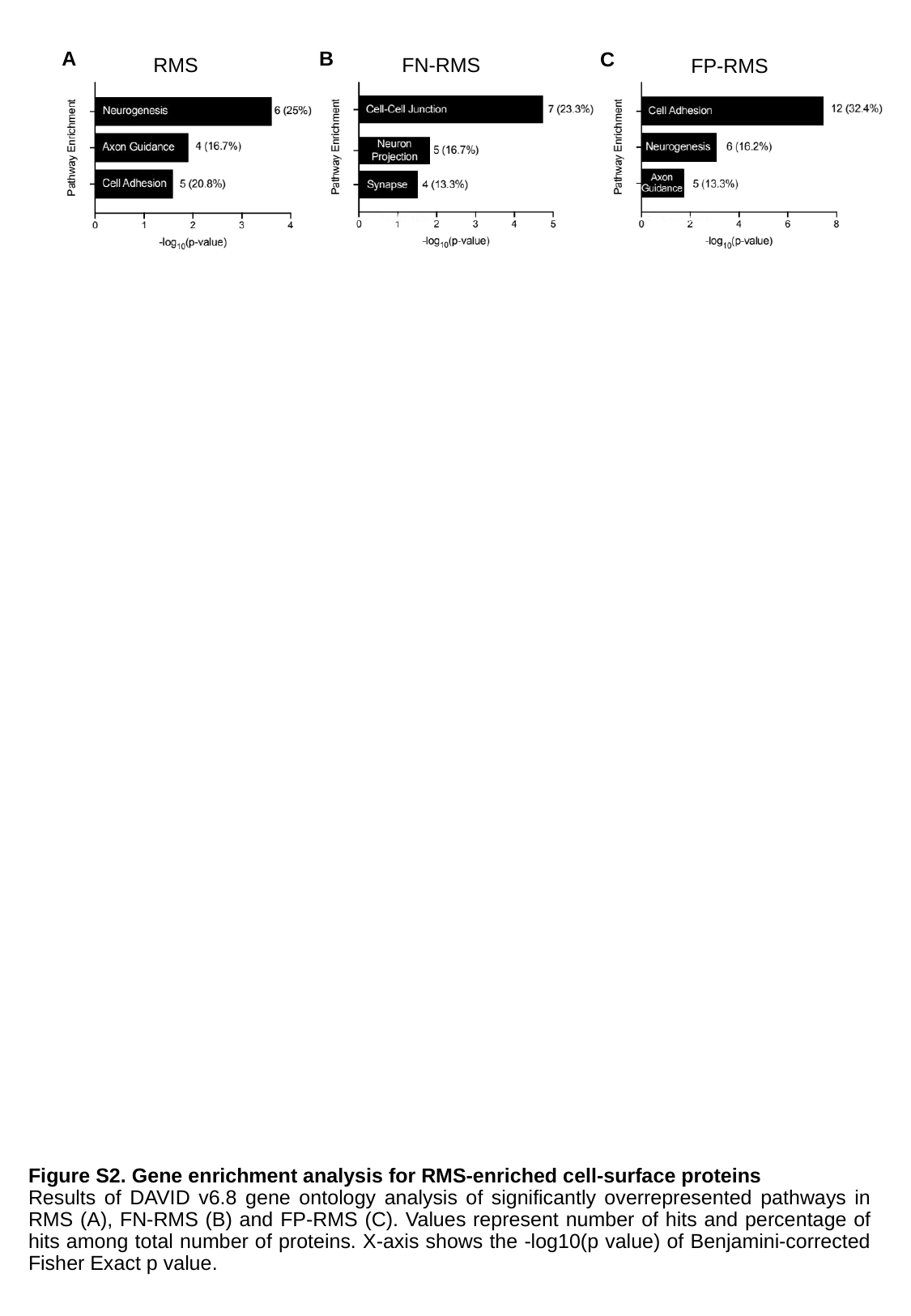

A
B
C
RMS
FN-RMS
FP-RMS
Figure S2. Gene enrichment analysis for RMS-enriched cell-surface proteins
Results of DAVID v6.8 gene ontology analysis of significantly overrepresented pathways in RMS (A), FN-RMS (B) and FP-RMS (C). Values represent number of hits and percentage of hits among total number of proteins. X-axis shows the -log10(p value) of Benjamini-corrected Fisher Exact p value.

## Slide 3
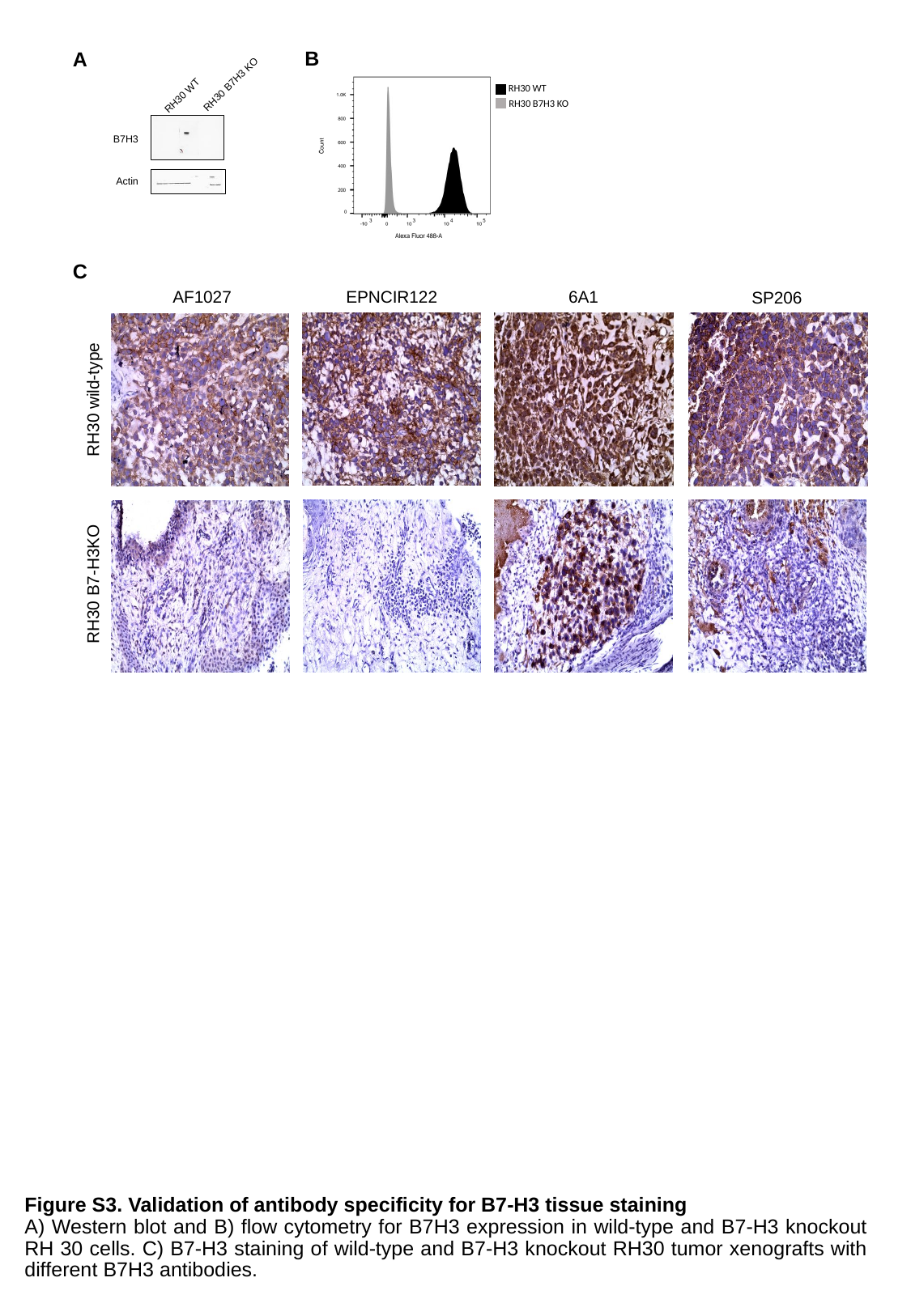

B
A
RH30 B7H3 KO
RH30 WT
RH30 WT
RH30 B7H3 KO
B7H3
Actin
C
EPNCIR122
6A1
AF1027
SP206
RH30 wild-type
RH30 B7-H3KO
Figure S3. Validation of antibody specificity for B7-H3 tissue staining
A) Western blot and B) flow cytometry for B7H3 expression in wild-type and B7-H3 knockout RH 30 cells. C) B7-H3 staining of wild-type and B7-H3 knockout RH30 tumor xenografts with different B7H3 antibodies.
