# Supplementary material for: Surfaceome Profiling of Rhabdomyosarcoma Reveals B7-H3 as a Mediator of Immune Evasion
Source: Cancers (Basel). 2021 Sep 9;13(18):4528. doi: 10.3390/cancers13184528 (PMC8466404; doi:10.3390/cancers13184528)
Supplement: Supplementary file 1 [file cancers-13-04528-s001.zip › Supplementary Materials.pdf]

Supplementary Materials

Materials and Methods

Mass Spectrometry

Denaturated and reduced proteins were run in an SDS-PAGE electrophoresis gel. Bio-Rad Bio-Safe Commassie stained gel bands were destained in 50% acetonitrile/50mM Tris pH 8.2 until clear. Proteins were digested in-situ with 0.125ug trypsin (Promega Corporation) in 25 mM Tris pH 8.2 / 0.0002% Zwittergent 3-16, at 37°C overnight, followed by peptide extraction with 2% trifluoroacetic acid and acetonitrile. The pooled extracts were concentrated, and the proteins were identified by nano-flow liquid chromatography electrospray tandem mass spectrometry (nanoLC-ESI-MS/MS) using a Thermo Scientific Q-Exactive Mass Spectrometer (Thermo Fisher Scientific) coupled to a Thermo Ultimate 3000 RSLCnano HPLC system. The digested peptide mixture was loaded onto a Halo C18 2.7µm EXP stem trap (Optimize Technologies) and chromatography was performed using 0.2 % formic acid in both the A solvent (98%water/2%acetonitrile) and B solvent (80% acetonitrile/10% isopropanol/10% water), with a 5%B to 40%B gradient over 110 minutes at 400 nl/min through a PicoFrit (New Objective, Woburn, MA) 100um x 35cm column handpacked with Agilent Poroshell 1.9um 120 EC C18 packing. The Q-Exactive mass spectrometer experiment was a data-dependent set up with an MS1 survey scan from 340-1500 m/z at resolution 70,000 (at 200m/z), followed by HCD MS/MS scans on the top 15 ions having a charge state of +2, +3, or +4, at resolution 17,500. The ions selected for MS/MS were placed on an exclusion list for 30 seconds. The MS1 AGC target were set to 1e6 and the MS2 target to 1e5 with max ion inject times of 50ms for both.

Tandem mass spectra were extracted by msconvert version 3.0.9134. All MS/MS samples were analyzed using Mascot (Matrix Science; version 2.4.0) and X! Tandem (The GPM, thegpm.org; version X! Tandem Sledgehammer (2013.09.01.1)). Mascot and X! Tandem were set up to search a current Swissprot human database with reverse decoy (40990 entries) assuming the digestion

enzyme stricttrypsin and with a fragment ion mass tolerance of 0.020 Da and a parent ion tolerance of 10.0 PPM. Glu->pyro-Glu of the n-terminus, ammonia-loss of the n-terminus, gln->pyro-Glu of the n-terminus, oxidation of methionine is specified in X! Tandem as variable modifications and carbamidomethyl of cysteine were specified as a fixed modification. Oxidation of methionine and carbamidomethyl of cysteine were specified in Mascot as variable modifications and fixed modifications, respectively.

Scaffold (version Scaffold_4.8.3, Proteome Software Inc.) was used to validate MS/MS-based peptide and protein identifications. Peptide identifications were accepted if they can be established at greater than 95.0% probability by the Scaffold Local FDR algorithm. Protein identifications were accepted if they can be established at greater than 95.0% probability and contain at least five identified peptides. Using these criteria, the protein FDR was 0.9%. Protein probabilities were assigned by the Protein Prophet algorithm[54]. Proteins that contain similar peptides and cannot be differentiated based on MS/MS analysis alone were grouped to satisfy the principles of parsimony. Proteins sharing significant peptide evidence were grouped into clusters. Spectral counts were normalized using the Normalized Spectral Abundance Factor (NSAF) as previously described[55]. NSAF values were used to identify proteins upregulated (fold change ≥ 2) in fusion-negative and fusion-positive RMS cell lines compared to normal skeletal muscle.

Western blot

Whole-cell extracts were lysed by radioimmunoprecipitation assay buffer (Thermo Scientific, 88901) containing protease inhibitors (Thermo Scientific, 1860932) and protein concentration was determined using BCA assay (Thermo Scientific, 23228). Immunoblotting was performed using a 4-12% gradient precast gel (Invitrogen, NP0321BOX) and nitrocellulose membrane using a semi-dry blotting system (Invitrogen, IB23002). The membrane was blocked in TBS containing 3% BSA for 1h at RT and incubated with rabbit anti-B7H3 (1:1000, Cell Signaling, 14058S) and mouse

anti-actin (1:5000, Cell Signaling, 4970S) O/N at 4^{0}C. After washing the membrane with TBS/0.1%Tween-20, membrane was incubated with HRP-conjugated secondary antibody (1:10,000, Invitrogen, 65-6120). Protein detection was performed using chemiluminescent substrate (Thermo Scientific, 34580) and a Biorad ChemiDoc XRS scanner for chemiluminescence imaging.

Flow cytometry

After trypsinization, cells were washed, and cell surface staining was performed for 30 min at 4°C. Antibodies were used following manufacturer's instructions; anti-PD-L1 PE (BioLegend, 329706), anti-PD-L2 PE (BioLegend, 329605), anti-B7H3 PE (Biolegend, 331606), anti-B7H4 PE (BioLegend, 358103), anti-B7H6 AF647 (R&D, FAB71442R), anti-B7H7 AF647 (R&D, FAB80841R), mouse IgG2b PE (Bilogend. 402204), mouse IgG2a PE (R&D, IC003P), mouse IgG1 PE (BioLegend, 400112) and mouse IgG1 APC (BioLegend, 400122). Cells were washed twice, fix in PFA 1% and analyzed in CantoX flow cytometer (Becton Dickinson) applying the same voltage for detection of PE or AF647 across all samples.

Immunohistochemistry

Paraffin-embedded tissue slides of fusion-positive (n=37) and fusion-negative (n=95) RMS tumors were obtained from resected specimens and biopsies in our institution (IRB #19-012426). Tissue sections were deparaffinized, rehydrated, and heated in antigen retrieval citrate solution, pH 6. Sections were incubated with hydrogen peroxidase blocking reagent (Abcam, ab64218) and were blocked with BSA 3% + goat serum 10%. Slides were then incubated at O/N at 4°C with anti-B7H3 antibody (1:1000, R&D System; AF1027), anti-MyoD1 (GenTex, GTX27272), or anti-Myogenin (1:1000, Abcam, ab1835) followed by biotinylated goat anti-mouse anti-rabbit (Abcam, ab64257), streptavidin peroxidase (Abcam, ab64269) and the diaminobenzidine (DAB) substrate (Abcam, ab64238). Slides were then counterstained with hematoxylin and mounded using DPX

Mountant (Sigma, 06522). B7-H3 H-score was calculated by an expert pathologist in a blinded manner. Tissue MicroArray containing a total of 99 cores of 32 types of normal human organs (Biomax, FDA999u) was stained following the same protocol. Optimization of B7H3 was performed using 4 different antibodies (R&D System; AF1027; Abcam, ab134161; Abcam, ab105922; Abcam, ab227670) on wild-type and B7H3KO RH30 tumor xenografts using the chorioallantoic membrane assay as previously described[56].
